# Effects of Oral Iron and Commercial Colostrum Supplementation on Clinical Health and Haematological Parameters in Neonatal Chianina Beef Calves: A Pilot Study

**DOI:** 10.3390/ani16193134

**Published:** 2026-10-06

**Authors:** Sala Giulia, Cinelli Vittoria, Minieri Sara, Meoli Gaia, Castelli Matteo, Wiedemann Ludovica, Sgorbini Micaela, Bonelli Francesca

**Affiliations:** 1 Department of Veterinary Medicine and Animal Science, University of Milan, 26900 Lodi, Italy; 2 Department of Veterinary Sciences, University of Pisa, 56124 Pisa, Italy; vittoriacinelli@yahoo.it (C.V.); sara.minieri@unipi.it (M.S.); ludovica.wiedemann@phd.unipi.it (W.L.); micaela.sgorbini@unipi.it (S.M.); francesca.bonelli@unipi.it (B.F.); 3 Department of Agriculture, Food, Environment and Forestry, University of Florence, 50144 Florence, Italy; gaia.meoli@unifi.it; 4 Vet’Haut-Pays Clinique Vétérinaire de Lumbres, 62380 Lumbres, France; mcastelli476@gmail.com

**Keywords:** Chianina cattle, beef calves, colostrum supplementation, iron supplementation, neonatal calf, haematology, calf health, passive transfer, extensive farming, pilot study

## Abstract

The neonatal period is a critical stage for calf health and development, and nutritional interventions have been proposed to support neonatal adaptation and reduce disease risk. However, evidence regarding their effectiveness in extensively reared beef breeds remains limited. This pilot study evaluated the effects of oral iron supplementation and administration of a commercial colostrum supplement on clinical health, haematological profile, passive transfer status, and growth performance in neonatal Chianina beef calves. Eighteen calves were randomly allocated to three groups: a control group, an oral iron supplementation group, and a commercial colostrum supplementation group. Clinical examinations and blood sampling were performed 48 h, 14 days, and 30 days after birth. Longitudinal data were analysed using linear mixed models and generalised estimating equations. No statistically significant overall treatment group effect was observed on the corrected Clinical Total Score (*p* = 0.123), although exploratory comparisons at the final assessment (30 days of age) showed lower scores in calves receiving the commercial colostrum supplement than in Control calves (nominal *p* = 0.008) and iron-supplemented calves (nominal *p* = 0.039). No significant differences were observed in growth performance, serum iron concentration, haematological variables, or the incidence of neonatal calf diarrhoea and bovine respiratory disease. Most haematological variables changed over time, reflecting normal neonatal physiological adaptation. These preliminary findings suggest a possible difference in clinical condition at 30 days of age in calves receiving commercial colostrum supplementation, but do not demonstrate an overall treatment effect during the study period. A single oral administration of the iron-containing supplement did not provide measurable clinical, haematological, or growth-related benefits under the conditions investigated.

## Simple Summary

The first weeks of life are critical for the health and development of beef calves. During this period, nutritional strategies such as oral iron supplementation and commercial colostrum supplementation have been proposed to support calf health; however, information on their effectiveness in extensively reared beef breeds remains limited. In this pilot study, we compared the effects of oral iron supplementation and a commercial colostrum supplement in newborn Chianina calves raised under extensive farming conditions. No statistically significant overall treatment effect was observed on the Clinical Total Score. At 30 days of age, however, calves receiving the colostrum supplement showed lower clinical scores than calves in the Control and Iron groups in exploratory comparisons. No significant differences were observed in growth, blood parameters, or the occurrence of diarrhoea and respiratory disease. Oral iron supplementation did not produce measurable clinical, haematological, or growth-related benefits under the conditions investigated. Because the study included a limited number of animals and was conducted on a single farm with a low incidence of disease, these findings should be considered preliminary. Larger studies are needed to determine whether the differences in clinical scores observed at 30 days of age reflect a clinically relevant benefit of colostrum supplementation.

## 1. Introduction

The neonatal period is one of the most critical stages in cattle production because health and growth during the first weeks of life strongly influence subsequent survival, welfare, and productive performance [1,2,3,4]. During this period, calves are particularly susceptible to infectious diseases, especially neonatal calf diarrhoea (NCD) and bovine respiratory disease (BRD), which remain among the leading causes of morbidity, mortality, and economic losses in beef and dairy production systems [5,6,7]. The occurrence of these diseases is multifactorial and is closely associated with inadequate passive transfer of immunity, nutritional deficiencies, and management practices during the first days of life [8,9].

Among nutritional factors influencing neonatal health, iron plays an essential role in erythropoiesis, oxygen transport, cellular metabolism, and immune function [9,10,11]. Newborn calves are particularly prone to iron deficiency because they are born with limited body iron reserves while bovine milk contains relatively low iron concentrations [12,13]. Consequently, iron deficiency has been associated with impaired haematological status, reduced growth performance, and increased susceptibility to infectious diseases [12,14]. Although iron supplementation has been proposed as a practical strategy to prevent these conditions, published studies have reported inconsistent results regarding its effects on calf health and haematological development [9,10,13].

Successful passive transfer of immunity through colostrum intake is another cornerstone of neonatal calf management. Besides immunoglobulins, colostrum contains numerous bioactive compounds, including growth factors, antimicrobial peptides, cytokines, hormones, and immune cells, which contribute to gastrointestinal development and immune maturation [15,16,17]. Adequate colostrum management, particularly regarding quality, quantity, and timing of administration, is therefore essential to reduce the risk of failure of passive transfer and subsequent neonatal disease [18,19,20]. Commercial colostrum supplements have increasingly been proposed as practical tools to support neonatal health, although evidence of their effectiveness under field conditions remains limited, particularly in beef production systems.

Most studies investigating iron supplementation and colostrum-derived products have been conducted in intensively managed dairy calves, whereas considerably less information is available for extensively reared beef breeds. This knowledge gap is particularly evident for autochthonous breeds such as Chianina, one of the most important Italian beef breeds, which is traditionally raised under extensive or semi-extensive conditions and represents an important component of the country’s livestock biodiversity [21,22,23]. Because management practices, environmental conditions, and disease pressure differ substantially between dairy and beef production systems, the effectiveness of nutritional supplementation strategies cannot be assumed to be the same across production contexts.

Therefore, the aim of the present study was to evaluate the effects of a commercial iron-containing nutritional supplement and administration of a commercial bovine colostrum supplement on growth performance, haematological profile, passive transfer status, and clinical health indicators in neonatal Chianina beef calves reared under extensive farming conditions. We hypothesised that calves receiving either iron supplementation or commercial colostrum supplementation would exhibit improved haematological status and better clinical health during the first month of life compared with untreated control calves.

## 2. Materials and Methods

### 2.1. Study Design and Animals

Given the limited available evidence in extensively reared beef calves, this study was designed as a prospective pilot field trial to generate preliminary evidence on the clinical and haematological effects of two neonatal supplementation strategies.

The study was conducted on a commercial Chianina beef cattle farm located in Rosignano Marittimo (Livorno, Central Italy). The herd consisted of approximately 60 purebred Chianina cattle managed under a closed extensive production system, with no introduction of replacement animals from external farms.

The production system included breeding cows, replacement heifers, fattening bulls slaughtered at approximately 20 months of age, and one breeding bull. During the winter season (November to March), animals were housed in a barn with free access to an outdoor paddock, whereas during spring and summer cows grazed on adjacent pastures where natural mating occurred.

Approximately one week before the expected calving date, pregnant cows were transferred to individual straw-bedded maternity pens. Following parturition, calves remained with their dams throughout the experimental period and suckled maternal colostrum and milk ad libitum. From the second week of life onward, calves were gradually exposed to hay and total mixed ration (TMR) to promote rumen development.

The experimental protocol was approved by the Animal Welfare Committee of the University of Pisa (OPBA, Protocol No. 67/2024; 12 December 2024).

### 2.2. Experimental Design

Eighteen Chianina calves of both sexes were enrolled immediately after birth. To be included, calves should: (1) be purebred singleton Chianina calves born at term following an uncomplicated calving, (2) be viable and clinically healthy based on a clinical examination performed by the veterinarian (VC) at birth, (3) remain with their dams, (4) and have unrestricted access to maternal colostrum. Calves were excluded in cases of prematurity, twin birth, dystocia requiring major obstetrical assistance, congenital abnormalities, severe neonatal disease, inability to suckle, or failure to complete the scheduled treatment and follow-up procedures.

Eligible calves were randomly assigned to three treatment groups (*n* = 6 calves each) using a computer-generated randomization list at birth. Calves included to the Control group (CG), comprising two males and four females, received no nutritional supplementation and were managed according to the routine farm protocol. Calves assigned to the Iron group (IG), comprising one male and five females, received a single oral administration (12 mL) of an iron supplement (Boviferm Primo^®^, Zac S.r.l., Turin, Italy) at approximately 24 h after birth. Each dose contained vitamin A (2,496,000 IU), vitamin C (2466 mg), vitamin D3 (2400 IU), vitamin E (2496 mg), β-carotene (150 mg), chelated iron (99.9 mg), selenium (0.50 mg as sodium selenite), and *Enterococcus faecium* DSM 10663 NCIMB 10415 (ORALIN^®^, Chevita Tierarzneimittel GmbH, Hengersberg, Germany; 3.6 × 10^9^ CFU). The administered dose was selected according to the manufacturer’s recommendations. The 24 h time point was predefined in the experimental protocol to ensure standardised supplementation during the early postnatal period, after the initial period of maternal colostrum intake, consistent with previous studies in which oral iron supplementation was initiated during the first day of life [24].

Calves assigned to the Colostrum group (ColG), included three males and three females, received a single oral administration of 100 mL of a commercial bovine colostrum supplement (Biocolost B^®^, Zac S.r.l., Turin, Italy) within the first 8 h of life, in addition to unrestricted suckling of maternal colostrum. The administered dose was selected according to the manufacturer’s recommendations, while the timing was selected to provide the supplement during the early postnatal period, when intestinal permeability to colostral immunoglobulins is still relatively high and before the progressive decline in IgG absorption associated with gut closure [25]. Each bottle contained 2 g of bovine colostral immunoglobulins. Since calves remained with their dams and were allowed unrestricted suckling, the individual amount of maternal colostrum consumed by each calf could not be quantified.

Each calf underwent clinical and laboratory evaluations at three predefined sampling times: 48 h after birth (T1), selected to allow completion of intestinal immunoglobulin absorption and stabilisation of early neonatal physiological changes; 14 days of age (T2), representing the period of transition from immediate neonatal adaptation to active postnatal development; and 30 days of age (T3), allowing evaluation of the persistence of treatment effects on clinical status, haematological profile, and growth during the first month of life.

### 2.3. Blood Sampling

Blood samples were collected from the jugular vein by the same operator (VC) at each sampling time (T1, T2, and T3). At each sampling, blood was collected into two 6 mL Vacu-Med^®^ tubes (FL Medical, Torreglia, Italy): one K2-EDTA tube for haematological analyses and one plain tube without additives for serum analyses.

Immediately after collection, all samples were stored in a refrigerated thermal container (approximately 4 °C) and transported to the Laboratory of Veterinary Clinical Pathology, Department of Veterinary Sciences, University of Pisa (UNI EN ISO 9001:2015). EDTA samples were analysed within 2 h of collection. Serum samples were centrifuged using a Legend RT centrifuge (Sorvall; Thermo Fisher Scientific Inc., Waltham, MA, USA) at 900× *g* for 10 min at 20 °C before analyses.

### 2.4. Laboratory Analyses

Complete blood count (CBC) was performed using a ProCyte Dx^®^ haematology analyser (IDEXX Laboratories Inc., Westbrook, ME, USA), based on laminar flow impedance technology. The analysis included red blood cell count (RBC), haematocrit (HCT), haemoglobin concentration (HGB), mean corpuscular volume (MCV), mean corpuscular haemoglobin (MCH), mean corpuscular haemoglobin concentration (MCHC), red cell distribution width (RDW), reticulocyte count, total and differential leukocyte count, platelet count, mean platelet volume (MPV), platelet distribution width (PDW), and platelet (PCT). Instrument quality control was performed daily according to the manufacturer’s recommendations.

Serum total protein (TP) concentration was determined by refractometry using a veterinary refractometer (Virbac, Carros, France) at T1 and used as an indirect indicator of passive transfer of immunity.

Serum iron concentration was measured at each sampling time using a SAT 450 automated clinical chemistry analyser (AMS Alliance, Rome, Italy; distributed by Assel S.r.l., Guidonia Montecelio, Italy) with the FERRO F colorimetric assay based on the Ferene method.

### 2.5. Clinical Examination and Clinical Scoring

After blood collection, each calf has been clinically evaluated by the same operator (VC), who was blinded to treatment allocation. During these procedures, calves were only manually restrained. A complete physical examination was performed by evaluating general health status, hydration, mucous membrane appearance, capillary refill time, superficial lymph nodes, heart rate, respiratory rate, rectal temperature, and thoracic auscultation. Body weight was estimated using a validated calibrated weight tape (Animeter^®^, Kerbl GmbH, Buchbach, Germany).

Calf health was assessed using two validated clinical scoring systems. Respiratory and general health were evaluated using the Wisconsin Calf Health Scoring Chart (University of Wisconsin School of Veterinary Medicine) [26], which assigns scores ranging from 0 (normal) to 3 (severely abnormal) for nasal discharge, ocular discharge, ear position, cough, rectal temperature, faecal consistency, umbilical status, and joint abnormalities. Neonatal vitality was assessed according to the scoring system described by Boccardo et al. (2019) [27], including vigour (score 1–5) and suckle reflex (score 0–3). The Clinical Total Score was calculated as the sum of the scores assigned to nasal discharge, ocular discharge, ear position, cough, rectal temperature, faecal consistency, umbilical status, joint abnormalities, vitality, and suckle reflex. As the original vitality score ranged from 1 (severely impaired vitality) to 5 (optimal vitality), it was reverse-coded as 5 minus the original score before inclusion in the composite score. Neonatal calf diarrhoea and bovine respiratory disease were diagnosed according to the predefined thresholds of the adopted scoring system: NCD was diagnosed when the faecal consistency score was ≥ 2; BRD was diagnosed when at least two of the four respiratory categories had a score ≥ 2, according to the predefined thresholds of the Wisconsin Calf Health Scoring Chart [26].

### 2.6. Statistical Analysis

An a priori sample size calculation was performed using G*Power software (version 3.1), considering a repeated-measures ANOVA with a within–between interaction. The calculation assumed three treatment groups, three repeated measurements, a large effect size (f = 0.50), a significance level of 0.05, a desired statistical power of 80%, a correlation of 0.50 among repeated measurements, and a nonsphericity correction factor (ε) of 1. Under these assumptions, the minimum required sample size was 12 calves (four per group), corresponding to an actual calculated power of 86.2%. A total of 18 calves (six per group) were enrolled in the study.

Statistical analyses were performed using IBM SPSS Statistics version 30 (IBM Corp., Armonk, NY, USA).

Data distribution was assessed using the Shapiro–Wilk test. Continuous variables are presented as median and interquartile range (IQR), whereas categorical variables are reported as absolute frequencies and percentages.

Comparisons among treatment groups at the first post-treatment assessment (T1, 48 h after birth) were performed using the Kruskal–Wallis test. When appropriate, pairwise post hoc comparisons were adjusted using the Bonferroni correction.

Longitudinal continuous outcomes were analysed using linear mixed-effects models. Treatment group, sampling time, and their interaction were included as fixed effects, whereas calf identification was included as a random intercept to account for repeated measurements within animals. Sampling time was specified as the repeated factor using a diagonal covariance structure.

Because serum total protein concentration was measured only to evaluate passive transfer at the first sampling time (T1), this variable was analysed exclusively at T1 and was not included in the longitudinal models.

Clinical scores were analysed using generalised estimating equations (GEE). For the GEE analyses performed in SPSS, the GENLIN procedure was used, specifying calf identification as the repeated subject and an exchangeable working correlation structure to account for repeated measurements within calves. Overall clinical score and respiratory score were modelled assuming a Poisson distribution with a log-link function, whereas the presence of altered faeces (faecal score ≥ 1) was analysed using a binomial distribution with a logit-link function. Treatment group, sampling time and their interaction were included as explanatory variables, and calf identification was specified as the repeated subject using an exchangeable working correlation matrix.

Statistical significance was set at *p* < 0.05. Given the exploratory nature of this pilot study, no formal adjustment for multiple testing across outcomes was applied. Accordingly, *p*-values are reported as nominal values, and statistically significant findings were interpreted cautiously.

## 3. Results

### 3.1. Study Population

A total of 18 Chianina calves were enrolled in the study and completed the experimental protocol, with six calves assigned to each treatment group. Clinical and laboratory evaluations were successfully performed at all three scheduled sampling times (T1, T2, and T3), yielding a total of 54 observations for longitudinal analyses. Descriptive statistics for all clinical, biochemical, and haematological parameters assessed at the first post-treatment evaluation (T1), stratified by treatment group, are provided in Supplementary Table S1.

At the first post-treatment assessment (T1, 48 h after birth), no statistically significant differences were detected among treatment groups in age, body weight, vital parameters, serum total protein concentration, serum iron concentration, or the haematological variables reported in Table 1. However, as T1 measurements were obtained after the administration of the experimental supplements, these findings cannot be considered evidence of group comparability before treatment.

### 3.2. Clinical Outcomes

The incidence of clinical disease during the study period was 27.8% (5/18 calves) for the NCD and 16.7% (3/18 calves) for BRD, with cases occurring sporadically across all treatment groups and without an evident clustering pattern.

Clinical scores remained generally low throughout the study. Ocular discharge was not observed in any calf, whereas nasal discharge, cough, ear position, joint abnormalities, and suckle reflex abnormalities occurred only sporadically and were therefore evaluated descriptively. Descriptive statistics for the corrected Clinical Total Score and its individual components, stratified by treatment group and sampling time, are provided in Supplementary Table S2.

Generalised estimating equation analysis of the corrected Clinical Total Score did not identify a statistically significant marginal effect of treatment group (*p* = 0.123) or sampling time (*p* = 0.464), or a significant treatment-by-time interaction (*p* = 0.757) (Table 2). At the final assessment (T3), exploratory pairwise comparisons showed lower Clinical Total Scores in the Colostrum group than in the Control group (Exp(B) = 0.500; 95% CI: 0.299–0.837; nominal *p* = 0.008) and the Iron group (Exp(B) = 0.565; 95% CI: 0.329–0.972; nominal *p* = 0.039). No statistically significant difference was detected between the Iron and Control groups (*p* = 0.545) (Table 3). These time-specific comparisons should be interpreted cautiously given the exploratory nature of the study, the small sample size, and the multiple comparisons performed.

Respiratory score was not influenced by treatment group (*p* = 0.824), sampling time (*p* = 0.652), or their interaction (*p* = 0.604). Likewise, the probability of altered faecal consistency did not differ among treatment groups (*p* = 0.565) or across sampling times (*p* = 0.383) (Table 2 and Table 3).

### 3.3. Growth Performance

Body weight increased significantly throughout the experimental period in all treatment groups (*p* < 0.001), reflecting normal postnatal growth. However, neither treatment group (*p* = 0.896) nor the interaction between treatment and sampling time (*p* = 0.731) significantly influenced body weight development (Table 4).

### 3.4. Serum Iron and Haematological Variables

Serum iron concentration varied significantly over time (*p* = 0.017). Although serum iron concentrations were descriptively higher in the Iron group at T1, no statistically significant treatment group effect (*p* = 0.204) or treatment-by-time interaction (*p* = 0.064) was detected (Table 4). Therefore, the observed between-group differences should be interpreted cautiously and cannot be considered evidence of a treatment-related effect.

Similarly, no significant treatment effects were detected for erythrogram variables. Haematocrit, haemoglobin concentration, mean corpuscular volume, mean corpuscular haemoglobin, mean corpuscular haemoglobin concentration, red cell distribution width, and absolute reticulocyte count changed significantly over time (all *p* < 0.05), whereas neither treatment group nor the treatment-by-time interaction significantly influenced any erythrocyte parameter (Table 4). The distributions of haematocrit and haemoglobin concentration across treatment groups and sampling times are illustrated in Supplementary Figure S1A,B.

Leukocyte variables followed the expected age-related physiological changes during the neonatal period. Significant temporal effects were observed for neutrophil percentage, lymphocyte percentage, monocyte percentage, eosinophil percentage, and the corresponding absolute cell count (*p* < 0.05). However, no significant effects of treatment group or treatment-by-time interaction were detected for any leukocyte variable (Table 4).

Platelet count also increased significantly over time (*p* < 0.001), whereas mean platelet volume remained stable throughout the study period (*p* = 0.340). Neither platelet variable was significantly affected by treatment group or by the interaction between treatment and sampling time (Table 4). Descriptive statistics for body weight and all laboratory variables included in the longitudinal analyses, stratified by treatment group and sampling time, are provided in Supplementary Table S3.

## 4. Discussion

The present pilot study explored the potential effects of two neonatal nutritional supplementation strategies—oral iron supplementation and administration of a commercial colostrum supplement—on clinical health, haematological profile, passive transfer status, and growth performance in neonatal Chianina beef calves reared under extensive farming conditions. To the authors’ knowledge, this is among the first prospective field studies comparing these two supplementation strategies in an autochthonous beef breed managed under an extensive production system. Given the exploratory nature of the study, the limited number of enrolled calves, and the low incidence of clinical disease observed throughout the experimental period, the findings should be considered preliminary and interpreted with caution. No statistically significant overall treatment group effect was observed on the corrected Clinical Total Score. Nevertheless, exploratory comparisons at the final assessment (T3) showed lower scores in calves receiving the commercial colostrum supplement than in those assigned to the Control and Iron groups. No treatment-related differences were detected in growth performance, serum iron concentration, or haematological variables. Most laboratory parameters changed significantly over time, consistent with the physiological adaptations that normally occur during the neonatal period rather than with treatment effects.

The lower Clinical Total Scores observed in calves receiving the commercial colostrum supplement at the final assessment may suggest a possible difference in clinical condition among treatment groups. However, this finding should be interpreted cautiously, as neither the overall treatment group effect nor the treatment-by-time interaction was statistically significant.

Furthermore, the differences observed in the exploratory pairwise comparisons at T3 were based on a limited number of animals and were not accompanied by significant differences in the incidence of neonatal calf diarrhoea or bovine respiratory disease. The low frequency of clinical events and the limited sample size may have reduced the ability to detect differences in disease occurrence. Therefore, the present findings cannot establish a clinically relevant benefit of commercial colostrum supplementation, but they may provide preliminary information for the design of future studies investigating its potential effects on calf health. From a biological perspective, the absence of a statistically significant treatment-by-time interaction suggests that the available data do not provide sufficient evidence of different clinical score trajectories among treatment groups during the first month of life. Although lower Clinical Total Scores were observed in the Colostrum group at T3, this time-specific finding was not accompanied by evidence of a differential longitudinal response to supplementation. Given the small sample size and the low frequency of clinical abnormalities, subtle differences in the temporal evolution of clinical health cannot be excluded.

Bovine colostrum contains numerous bioactive compounds, including lactoferrin, cytokines, growth factors, antimicrobial peptides, oligosaccharides, hormones, and immune cells, which contribute to intestinal maturation and modulation of both innate and adaptive immune responses [15,16,17]. These components provide a biological rationale for investigating whether colostrum supplementation may influence neonatal clinical health beyond the transfer of immunoglobulins. However, the exploratory differences in Clinical Total Score observed at T3 in the present study do not establish that these components were responsible for the findings. Moreover, serum total protein concentrations at T1 did not differ among treatment groups (*p* = 0.819), despite the commercial colostrum supplement having already been administered before sampling. Therefore, the present data do not provide evidence that supplementation improved passive transfer of immunity. The potential contribution of colostral bioactive components to the observed clinical findings remains uncertain and requires further investigation.

Previous studies have consistently highlighted the importance of appropriate colostrum management for reducing neonatal morbidity and improving calf performance [20,28]. However, most available evidence concerns dairy production systems, whereas considerably less information is available for extensively reared beef calves. Differences in management, environmental exposure, maternal behaviour, and disease pressure between dairy and beef production systems may substantially influence the response to neonatal nutritional interventions. Therefore, extrapolation of results obtained in dairy calves to beef breeds should be made cautiously. In this context, the present study provides preliminary evidence specifically generated under extensive beef farming conditions, contributing to the limited knowledge currently available for Chianina cattle.

Unlike commercial colostrum supplementation, oral iron administration did not significantly influence serum iron concentration, erythrogram variables, or growth performance. Although serum iron concentrations were descriptively higher in the supplemented group at the first evaluation, this difference was not maintained throughout the study and did not translate into measurable haematological or clinical benefits. Several factors may explain these findings. First, the iron status of the calves before supplementation was not assessed, and the potential influence of pre-existing individual differences on the response to treatment cannot be excluded. Second, the supplementation protocol consisted of a single oral administration, whereas studies reporting beneficial effects have generally adopted repeated supplementation schedules or parenteral administration, resulting in greater iron bioavailability [10,13].

The present findings are in agreement with those reported by Sickinger et al. (2024) [9], who did not observe significant improvements in the principal haematological variables following iron supplementation. Conversely, Mohri et al. (2004) [10] described significant increases in haemoglobin concentration, haematocrit, and erythrocyte count in supplemented calves. These apparently conflicting results may largely reflect differences in supplementation protocols, dosage, route of administration, baseline iron status, and production systems. Therefore, the absence of measurable benefits observed in the present study should not be interpreted as evidence that iron supplementation is universally ineffective but rather that the specific protocol adopted under the investigated management conditions did not produce detectable clinical or haematological improvements during the first month of life.

Several haematological variables changed significantly over time independently of treatment. Haematocrit, haemoglobin concentration, mean corpuscular volume, mean corpuscular haemoglobin, reticulocyte count, leukocyte populations, and platelet count all showed significant temporal variation. These findings are consistent with the physiological haematological maturation previously described in healthy neonatal calves, including the progressive replacement of foetal erythrocytes and the postnatal development of the immune system [29]. Consequently, the observed longitudinal changes most likely reflected normal developmental processes rather than the effects of nutritional supplementation.

Similarly, body weight increased progressively in all experimental groups, confirming normal postnatal growth. However, no significant differences among treatments were detected. Previous studies have reported improvements in average daily gain associated with successful passive transfer or iron supplementation [13,30], whereas others have reported limited or inconsistent effects. In the present study, the relatively short follow-up period, the low incidence of disease, and the generally good health status of the calves may have reduced the likelihood of detecting treatment-related differences in growth performance.

The findings of this study should be interpreted in light of several limitations. First, although an a priori sample size calculation was performed, the relatively small sample size (*n* = 6 calves per treatment group) may have limited the statistical power to detect small or moderate treatment effects, particularly for outcomes characterised by a low frequency of clinical events. The sample size calculation was based on a large assumed effect size and a repeated-measures group-by-time interaction and therefore does not guarantee adequate statistical power for all the clinical, haematological, and biochemical outcomes investigated. Second, the study was conducted on a single commercial farm with good management practices and low infectious pressure, limiting the generalizability of the findings to farms operating under different environmental or management conditions. Finally, calves were followed only during the first month of life, preventing assessment of possible long-term effects of neonatal supplementation on subsequent health, growth, or productive performance. A further limitation is that the iron-containing supplement evaluated in this study also provided vitamins, selenium and probiotic components, preventing attribution of the observed responses exclusively to iron supplementation. Moreover, a true pre-treatment blood sample was not collected, preventing direct assessment of baseline haematological and biochemical status and confirmation of group comparability before supplementation. Baseline iron status could therefore not be comprehensively characterised, also because serum ferritin and other indicators of body iron stores were not measured. Although calves were randomly allocated to treatment groups at birth, pre-existing individual differences cannot be excluded, particularly given the small sample size. Consequently, between-group differences should be interpreted cautiously, as treatment-related effects cannot be unequivocally distinguished from potential pre-existing differences. Likewise, the quality and immunoglobulin content of maternal colostrum were not directly evaluated, which may have influenced the response to supplementation. In addition, all calves received maternal colostrum ad libitum, while calves in the ColG group received the commercial colostrum supplement in addition to maternal colostrum. Therefore, total colostrum and IgG intake could not be standardised across groups, representing a potential confounding factor when interpreting the clinical effects observed in the ColG group.

Despite these limitations, the study provides preliminary information on neonatal nutritional supplementation in an autochthonous beef breed reared under extensive farming conditions, a production system that remains underrepresented in the scientific literature. These findings may therefore contribute to the design of larger multicentre studies aimed at clarifying the role of neonatal nutritional interventions in beef cattle.

Overall, under the management conditions investigated, no statistically significant overall treatment group effect was observed on the corrected Clinical Total Score. Nevertheless, exploratory comparisons at the final assessment (T3) showed lower scores in calves receiving the commercial colostrum supplement than in those assigned to the Control and Iron groups. A single administration of the iron-containing nutritional supplement was not associated with detectable improvements in the investigated clinical or haematological outcomes. Given the limited sample size, the low incidence of clinical disease, and the multiple statistical comparisons performed, these findings should be considered preliminary. Further studies involving larger populations, multiple farms, and longer follow-up periods are needed to determine whether the differences observed at T3 are reproducible and clinically relevant.

## 5. Conclusions

This pilot study provides preliminary information on the effects of two neonatal nutritional supplementation strategies in Chianina beef calves reared under extensive farming conditions, a production system for which scientific evidence remains limited. No statistically significant overall treatment group effect was observed on the corrected Clinical Total Score. However, exploratory pairwise comparisons at 30 days of age showed lower Clinical Total Scores in calves receiving the commercial bovine colostrum supplement than in those assigned to the Control and Iron groups. No significant treatment-related differences were detected in growth performance, serum iron concentration, haematological variables, or the incidence of neonatal calf diarrhoea and bovine respiratory disease. A single administration of the commercial iron-containing nutritional supplement was not associated with detectable benefits in the outcomes investigated.

Given the exploratory nature of the study, the limited sample size, the single-farm design, and the low incidence of clinical disease, these findings should be interpreted cautiously. The differences observed in Clinical Total Score at the final assessment do not establish the clinical efficacy of colostrum supplementation. Larger multicentre studies, involving different management conditions and longer follow-up periods, are needed to determine whether these preliminary observations are reproducible and clinically relevant.

## Figures and Tables

**Table 1:** Kruskal–Wallis test results for comparisons among treatment groups at the first assessment (T1).

Variable	Kruskal–Wallis H	Kruskal–Wallis *p*-Value
Age, days	0.258	0.879
Body weight, kg	0.332	0.847
Heart rate, bpm	0.056	0.973
Respiratory rate, apm	3.430	0.180
Rectal temperature, °C	2.106	0.349
Total protein, g/dL	0.398	0.819
Serum iron, mg/dL	5.178	0.075
RBC, 1012/L	0.433	0.805
HCT, %	0.056	0.973
HGB, g/dL	0.222	0.895
MCV, fL	0.904	0.636
MCH, pg	0.904	0.636
MCHC, g/dL	0.247	0.884
RDW, %	0.114	0.945
Reticulocytes, %	1.417	0.492
Reticulocytes, K/µL	3.079	0.214
Leukocytes, 109/L	1.977	0.372
Neutrophils, %	1.310	0.519
Lymphocytes, %	1.958	0.376
Monocytes, %	1.910	0.385
Eosinophils, %	1.536	0.464
Basophils, %	2.613	0.271
Neutrophils, 109/L	0.608	0.738
Lymphocytes, 109/L	1.906	0.385
Monocytes, 109/L	3.105	0.212
Eosinophils, 109/L	1.278	0.528
Basophils, 109/L	0.432	0.806
Platelets, K/µL	3.789	0.150
MPV, fL	0.803	0.669
PDW, fL	5.750	0.056

**Table 2:** Generalised estimating equation results for clinical outcomes.

Outcome	Model	Effect	*p*-Value
Clinical Total Score	Poisson GEE	Treatment group	0.123
Clinical Total Score	Poisson GEE	Time	0.464
Clinical Total Score	Poisson GEE	Group × time	0.757
Respiratory Score	Poisson GEE	Treatment group	0.824
Respiratory Score	Poisson GEE	Time	0.652
Respiratory Score	Poisson GEE	Group × time	0.604
Altered faecal consistency	Binomial GEE	Treatment group	0.565
Altered faecal consistency	Binomial GEE	Time	0.383

For altered faecal consistency, only the main effects of treatment group and time were included in the final model because the model including the group-by-time interaction was unstable due to the low number of events. Altered faecal consistency was defined as a faecal score ≥ 1.

**Table 3:** Parameter estimates from generalised estimating equation models for clinical outcomes.

Outcome	Comparison	B	95% CI for B	Exp(B)	95% CI for Exp(B)	*p*-Value
Clinical Total Score	Iron vs. Control	−0.123	−0.520 to 0.274	0.885	0.595–1.316	0.545
Clinical Total Score	Colostrum vs. Control	−0.693	−1.208 to −0.178	0.500	0.299–0.837	0.008
Clinical Total Score	Colostrum vs. Iron	−0.571	−1.113 to −0.028	0.565	0.329–0.972	0.039
Respiratory Score	Iron vs. Control	−0.074	−0.745 to 0.596	0.929	0.475–1.816	0.829
Respiratory Score	Colostrum vs. Control	−0.336	−1.020 to 0.347	0.714	0.360–1.415	0.335
Respiratory Score	Colostrum vs. Iron	−0.262	−0.750 to 0.225	0.769	0.472–1.253	0.292
Normal faecal consistency	Iron vs. Control	−0.028	−1.364 to 1.308	0.972	0.256–3.697	0.967
Normal faecal consistency	Colostrum vs. Control	0.637	−0.736 to 2.009	1.890	0.479–7.458	0.363
Normal faecal consistency	Colostrum vs. Iron	0.665	−0.699 to 2.029	1.944	0.497–7.608	0.339

B: regression coefficient; CI: confidence interval; Exp(B): exponentiated regression coefficient; T3: final assessment at 30 days of age. Clinical Total Score and Respiratory Score were analysed using Poisson GEE models with a log-link function, including treatment group, sampling time, and their interaction. The reported pairwise comparisons for these outcomes refer to T3. Normal faecal consistency (faecal score = 0) was analysed using a binomial GEE model with a logit-link function. Only the main effects of treatment group and sampling time were included in the final model because the model including their interaction was unstable due to the low number of events. Consequently, the reported comparisons for faecal consistency represent treatment group effects across the study period rather than time-specific comparisons. For the Poisson models, Exp(B) represents the ratio of expected scores between the compared groups, whereas for the binomial model it represents the odds ratio for normal faecal consistency. Confidence intervals were calculated using robust standard errors. The reported *p*-values are nominal.

**Table 4:** Linear mixed-model results for body weight and laboratory variables.

Variable	Treatment Group *p*-Value	Time *p*-Value	Group × Time *p*-Value
Body weight, kg	0.896	<0.001	0.731
Serum iron, mg/dL	0.204	0.017	0.064
HCT, %	0.896	<0.001	0.864
HGB, g/dL	0.809	<0.001	0.247
MCV, fL	0.926	<0.001	0.895
MCH, pg	0.749	<0.001	0.827
MCHC, g/dL	0.731	0.005	0.395
RDW, %	0.847	<0.001	0.712
Reticulocytes, K/µL	0.429	0.002	0.544
Neutrophils, %	0.534	<0.001	0.386
Lymphocytes, %	0.523	<0.001	0.442
Monocytes, %	0.638	<0.001	0.618
Eosinophils, %	0.576	0.024	0.544
Neutrophils, 10^9^/L	0.516	0.012	0.819
Lymphocytes, 10^9^/L	0.192	<0.001	0.412
Monocytes, 10^9^/L	0.693	<0.001	0.565
Eosinophils, 10^9^/L	0.328	0.027	0.353
Platelets, K/µL	0.273	<0.001	0.676
MPV, fL	0.955	0.340	0.988

Group, time, and group-by-time interaction were included as fixed effects; calf identification was included as a random intercept. Reticulocyte percentage was not included in the longitudinal models because the recorded values showed very limited variability (0.0% or 0.1%), with all measurements equal to 0.0% at T3. Absolute reticulocyte count (K/µL), which retained greater numerical variability, was therefore used for the longitudinal analysis.

## Data Availability

The datasets generated and analysed during the current study are available from the corresponding author upon reasonable request.

## Supplementary Materials

The following supporting information can be downloaded at: https://www.mdpi.com/article/10.3390/ani16193134/s1. Supplementary Table S1: Descriptive data at the first post-treatment assessment (T1). Values are median [Q1–Q3]; T1 = 48 h after birth; Supplementary Table S2: Clinical score components by treatment group and sampling time. Data are presented as median [Q1–Q3] (*n* = 6 calves per group and time). Clinical Total Score was recalculated after reversing vitality; suckle reflex is unchanged (0 = normal, higher values indicate greater impairment); Supplementary Table S3: Descriptive statistics for longitudinal outcomes; Supplementary Figure S1A: Haematocrit (HCT) in Chianina calves assigned to the Control, Iron, and Colostrum groups at 48 h (T1), 14 days (T2), and 30 days (T3) of age. Data are presented as median and interquartile range (*n* = 6 calves per group). Supplementary Figure S1B: Haemoglobin concentration (HGB) in Chianina calves assigned to the Control, Iron, and Colostrum groups at 48 h (T1), 14 days (T2), and 30 days (T3) of age. Data are presented as median and interquartile range (*n* = 6 calves per group).

## Abbreviations

The following abbreviations are used in this manuscript:

NCD	Neonatal calf diarrhoea
BRD	Bovine respiratory disease
TMR	Total mixed ration
CG	Control group
IG	Iron group
ColG	Colostrum group
T1	48 h after birth
T2	14 days of age
T3	30 days of age
CBC	Complete blood count
RBC	Red blood cell count
HCT	Haematocrit
HGB	Haemoglobin concentration
MCV	Mean corpuscular volume
MCH	Mean corpuscular haemoglobin
MCHC	Mean corpuscular haemoglobin concentration
RDW	Red cells distribution width
MPV	Mean platelet volume
PDW	Platelet distribution width
PCT	Platelet
TP	Serum total protein
IQR	Interquartile range
GEE	Generalised estimating equation
