# Supplementary material for: Effects of Oral Iron and Commercial Colostrum Supplementation on Clinical Health and Haematological Parameters in Neonatal Chianina Beef Calves: A Pilot Study
Source: Animals (Basel). 2026 Oct 6;16(19):3134. doi: 10.3390/ani16193134 (PMC13663556; doi:10.3390/ani16193134)
Supplement: Supplementary file 1 [file animals-16-03134-s001.zip › animals-4521570-supplementary.pdf]

**Supplementary Table S1**. Descriptive data at the first post-treatment assessment (T1)
Values are median [Q1–Q3]; T1 = 48 h after birth.

| Parameter | Control (n = 6) | Iron (n = 6) | Colostrum (n = 6) |
|---|---|---|---|
| **General clinical and biochemical parameters** | | | |
| Age, days | 1.50 [1–2] | 1.50 [1–2] | 1 [1–1.75] |
| Body weight, kg | 59.50 [55.75–61] | 57.50 [56.25–60.25] | 58.50 [55.75–59.75] |
| Heart rate, bpm | 166 [140–180] | 153 [138.50–178] | 160 [144–173] |
| Respiratory rate, apm | 56 [47.50–60] | 43 [37.50–56] | 44 [38–47] |
| Rectal temperature, °C | 39.10 [38.92–39.35] | 38.85 [38.65–38.98] | 39.05 [38.85–39.10] |
| Total protein, g/dL | 5.70 [5.25–6.30] | 5.70 [5.28–6.12] | 5.65 [5.28–5.95] |
| Serum iron, mg/dL | 89 [71–108] | 165 [131.25–198.50] | 50.50 [38.50–101.50] |
| **Erythrogram and reticulocytes** | | | |
| RBC, $10^{12}$/L | 7.26 [6.71–9.05] | 8.23 [7.02–8.96] | 8.02 [7.16–8.33] |
| HCT, % | 29.50 [28.28–40.33] | 35.40 [27.57–40.83] | 31.80 [29.30–32.35] |
| HGB, g/dL | 9 [8.65–11.90] | 10.60 [8.95–11.88] | 9.90 [9.15–9.97] |
| MCV, fL | 42.85 [40–44.58] | 41.85 [39.23–45.07] | 40.95 [39.48–41.67] |
| MCH, pg | 13.10 [12.22–13.45] | 13.05 [12.53–13.20] | 12.75 [12.10–12.88] |
| MCHC, g/dL | 30.55 [30.42–30.60] | 30.45 [29.32–31.43] | 30.75 [30.50–31.15] |
| RDW, % | 30.45 [28.48–31.23] | 31.20 [29.65–31.70] | 30.25 [28.80–32.60] |
| Reticulocytes, % | 0.05 [0–0.10] | 0 [0–0.08] | 0 [0–0] |
| Reticulocytes, K/μL | 2.95 [1.60–4.68] | 2.85 [2.52–4.75] | 1.25 [0.72–3.35] |
| **Leukogram** | | | |
| Leukocytes, $10^9$/L | 12.12 [9.66–13.22] | 10.16 [7.56–13.38] | 9.12 [8.52–9.57] |
| Neutrophils, % | 83.90 [62.75–87.20] | 83.80 [79.60–86.20] | 80.40 [79.08–81.42] |
| Lymphocytes, % | 13.75 [10.18–33.60] | 12.85 [10.25–15.53] | 16.45 [15.62–17.73] |
| Monocytes, % | 0.75 [0.23–2.25] | 0.15 [0.10–1.02] | 0.65 [0.60–0.93] |
| Eosinophils, % | 1.10 [0.30–1.60] | 0.30 [0.23–0.38] | 0.45 [0.25–0.50] |
| Basophils, % | 0.85 [0.50–0.90] | 0.80 [0.45–1.45] | 1.25 [0.82–1.68] |
| Neutrophils, $10^9$/L | 9.03 [5.51–11.56] | 8.73 [6.29–10.75] | 7.39 [6.76–7.70] |
| Lymphocytes, $10^9$/L | 1.81 [1.35–3.22] | 1.33 [1.25–1.37] | 1.42 [1.23–1.45] |
| Monocytes, $10^9$/L | 0.08 [0.04–0.20] | 0.02 [0.01–0.07] | 0.07 [0.05–0.09] |
| Eosinophils, $10^9$/L | 0.14 [0.02–0.23] | 0.04 [0.02–0.04] | 0.04 [0.03–0.06] |
| Basophils, $10^9$/L | 0.10 [0.04–0.13] | 0.10 [0.05–0.21] | 0.11 [0.07–0.20] |
| **Platelet parameters** | | | |
| Platelets, K/μL | 236.50 [180–279.50] | 213 [190.25–232] | 263.50 [253.25–283.50] |
| MPV, fL | 8.75 [8.55–9.25] | 9 [8.57–9.50] | 9.30 [8.88–9.65] |
| PDW, fL | 7.45 [7.28–7.62] | 7.10 [6.85–7.65] | 8.65 [8.30–8.83] |

**Supplementary Table S2.** Clinical score components by treatment group and sampling timeData are presented as median [Q1–Q3] (n = 6 calves per group and time). Clinical Total Score was recalculated after reversing vitality; suckle reflex is unchanged (0 = normal, higher values indicate greater impairment).

| Clinical parameter | Time | Control (n=6) | Iron (n=6) | Colostrum (n=6) |
|---|---|---|---|---|
| Clinical Total Score | T1 (48 h) | 2 [2–2.75] | 2 [2–3.5] | 2 [1.25–2.75] |
| | T2 (14 days) | 2 [2–5] | 2 [1.25–3.5] | 2 [1–3.75] |
| | T3 (30 days) | 5 [3.5–5] | 4 [2.5–4.75] | 2 [1–3] |
| Respiratory Score | T1 (48 h) | 2 [2–2] | 2 [1.25–2] | 2 [1.25–2] |

| | | | | |
|---|---|---|---|---|
| | T2 (14 days) | 1.5 [1–2] | 1.5 [1–2.75] | 1.5 [1–2] |
| | T3 (30 days) | 2 [1.25–3.5] | 2 [2–2] | 1.5 [1–2] |
| Nasal discharge | T1 (48 h) | 0 [0–0] | 0 [0–0] | 0 [0–0] |
| | T2 (14 days) | 0 [0–0] | 0 [0–0] | 0 [0–0] |
| | T3 (30 days) | 0 [0–0] | 0 [0–0] | 0 [0–0] |
| Ocular discharge | T1 (48 h) | 0 [0–0] | 0 [0–0] | 0 [0–0] |
| | T2 (14 days) | 0 [0–0] | 0 [0–0] | 0 [0–0] |
| | T3 (30 days) | 0 [0–0] | 0 [0–0] | 0 [0–0] |
| Ear position | T1 (48 h) | 0 [0–0] | 0 [0–0] | 0 [0–0] |
| | T2 (14 days) | 0 [0–0] | 0 [0–0] | 0 [0–0] |
| | T3 (30 days) | 0 [0–0] | 0 [0–0] | 0 [0–0] |
| Cough | T1 (48 h) | 0 [0–0] | 0 [0–0] | 0 [0–0] |
| | T2 (14 days) | 0 [0–0] | 0 [0–0] | 0 [0–0] |
| | T3 (30 days) | 0 [0–1.5] | 0 [0–0.75] | 0 [0–0] |
| Rectal temperature | T1 (48 h) | 2 [2–2] | 1.5 [1–2] | 2 [1.25–2] |
| | T2 (14 days) | 1.5 [1–2] | 1.5 [1–2] | 1.5 [1–2] |
| | T3 (30 days) | 2 [1.25–2] | 2 [1.25–2] | 1.5 [1–2] |
| Faecal consistency | T1 (48 h) | 0 [0–0] | 0.5 [0–1] | 0 [0–0] |
| | T2 (14 days) | 0 [0–0] | 0 [0–0] | 0 [0–0] |
| | T3 (30 days) | 1 [0.25–1] | 0 [0–0] | 0 [0–0] |
| Umbilical status | T1 (48 h) | 0 [0–0.75] | 0 [0–0] | 0 [0–0] |
| | T2 (14 days) | 0 [0–0.75] | 0 [0–0] | 0 [0–0] |
| | T3 (30 days) | 0 [0–0.75] | 0 [0–0] | 0 [0–0] |
| Joint abnormalities | T1 (48 h) | 0 [0–0] | 0 [0–0] | 0 [0–0] |
| | T2 (14 days) | 0 [0–0] | 0 [0–0] | 0 [0–0] |
| | T3 (30 days) | 0 [0–0] | 0 [0–0] | 0 [0–0] |
| Vitality | T1 (48 h) | 0 [0–0] | 0 [0–0] | 0 [0–0.75] |
| | T2 (14 days) | 0 [0–0] | 0 [0–0] | 0 [0–0] |
| | T3 (30 days) | 0 [0–0] | 0 [0–0] | 0 [0–0] |
| Suckle reflex | T1 (48 h) | 0 [0–0] | 0 [0–0] | 0 [0–0] |
| | T2 (14 days) | 0 [0–0.75] | 0 [0–0] | 0 [0–0] |
| | T3 (30 days) | 1 [0–2] | 0 [0–2.25] | 0 [0–0] |

**Supplementary Table S3**. Descriptive statistics for longitudinal outcomes

| Variable | Control | Iron | Colostrum |
|---|---|---|---|
| T1 – 48 h after birth | | | |
| Body weight, kg | 59.50 [55.75–61.00] | 57.50 [56.25–60.25] | 58.50 [55.75–59.75] |
| Serum iron, mg/dL | 89.00 [71.00–108.00] | 165.00 [131.25–198.50] | 50.50 [38.50–101.50] |
| HCT, % | 29.50 [28.28–40.33] | 35.40 [27.57–40.83] | 31.80 [29.30–32.35] |
| HGB, g/dL | 9.00 [8.65–11.90] | 10.60 [8.95–11.88] | 9.90 [9.15–9.97] |
| MCV, fL | 42.85 [40.00–44.58] | 41.85 [39.23–45.07] | 40.95 [39.48–41.67] |
| MCH, pg | 13.10 [12.22–13.45] | 13.05 [12.53–13.20] | 12.75 [12.10–12.88] |
| MCHC, g/dL | 30.55 [30.42–30.60] | 30.45 [29.32–31.43] | 30.75 [30.50–31.15] |
| RDW, % | 30.45 [28.48–31.23] | 31.20 [29.65–31.70] | 30.25 [28.80–32.60] |

| | | | |
|---|---|---|---|
| Reticulocytes, K/μL | 2.95 [1.60–4.68] | 2.85 [2.52–4.75] | 1.25 [0.72–3.35] |
| Neutrophils, % | 83.90 [62.75–87.20] | 83.80 [79.60–86.20] | 80.40 [79.08–81.42] |
| Lymphocytes, % | 13.75 [10.18–33.60] | 12.85 [10.25–15.53] | 16.45 [15.62–17.73] |
| Monocytes, % | 0.75 [0.23–2.25] | 0.15 [0.10–1.02] | 0.65 [0.60–0.93] |
| Eosinophils, % | 1.10 [0.30–1.60] | 0.30 [0.23–0.38] | 0.45 [0.25–0.50] |
| Neutrophils, $10^9$/L | 9.03 [5.51–11.56] | 8.73 [6.29–10.75] | 7.39 [6.76–7.70] |
| Lymphocytes, $10^9$/L | 1.81 [1.35–3.22] | 1.33 [1.25–1.37] | 1.42 [1.23–1.45] |
| Monocytes, $10^9$/L | 0.08 [0.04–0.20] | 0.02 [0.01–0.07] | 0.07 [0.05–0.09] |
| Eosinophils, $10^9$/L | 0.14 [0.02–0.23] | 0.04 [0.02–0.04] | 0.04 [0.03–0.06] |
| Platelets, K/μL | 236.50 [180.00–279.50] | 213.00 [190.25–232.00] | 263.50 [253.25–283.50] |
| MPV, fL | 8.75 [8.55–9.25] | 9.00 [8.57–9.50] | 9.30 [8.88–9.65] |
| T2 — 14 days of age | | | |
| Body weight, kg | 76.50 [70.75–77.00] | 74.50 [73.00–81.25] | 73.00 [70.00–73.75] |
| Serum iron, mg/dL | 105.50 [69.00–132.75] | 70.00 [47.00–94.25] | 45.50 [30.50–83.75] |
| HCT, % | 23.50 [21.52–35.45] | 27.05 [21.25–34.65] | 24.75 [22.50–26.40] |
| HGB, g/dL | 7.35 [6.80–10.68] | 8.95 [7.35–10.78] | 8.10 [7.32–8.57] |
| MCV, fL | 37.20 [36.60–40.88] | 37.70 [34.62–39.50] | 36.95 [35.62–38.27] |
| MCH, pg | 12.15 [11.10–12.67] | 12.25 [11.80–12.55] | 12.35 [11.85–12.47] |
| MCHC, g/dL | 30.75 [30.02–31.48] | 33.25 [31.57–34.40] | 32.55 [32.35–32.83] |
| RDW, % | 35.60 [34.52–36.60] | 36.65 [35.45–38.23] | 36.60 [36.35–36.85] |
| Reticulocytes, K/μL | 0.80 [0.17–1.65] | 0.70 [0.15–1.03] | 0.95 [0.12–1.77] |
| Neutrophils, % | 56.10 [50.42–58.55] | 56.20 [47.00–61.35] | 51.10 [48.40–56.50] |
| Lymphocytes, % | 39.30 [37.00–45.65] | 37.55 [33.27–43.62] | 44.85 [39.42–48.92] |
| Monocytes, % | 2.95 [2.58–3.10] | 2.55 [1.35–4.43] | 2.95 [1.38–3.33] |
| Eosinophils, % | 0.10 [0.10–0.18] | 0.20 [0.12–0.20] | 0.10 [0.10–0.18] |
| Neutrophils, $10^9$/L | 6.44 [5.77–6.85] | 6.05 [5.89–7.07] | 6.21 [4.57–7.36] |
| Lymphocytes, $10^9$/L | 4.66 [4.41–5.32] | 4.88 [3.67–6.76] | 5.00 [4.55–5.74] |
| Monocytes, $10^9$/L | 0.33 [0.28–0.35] | 0.41 [0.12–0.67] | 0.30 [0.14–0.40] |
| Eosinophils, $10^9$/L | 0.01 [0.01–0.02] | 0.02 [0.02–0.03] | 0.01 [0.01–0.02] |
| Platelets, K/μL | 625.50 [508.25–908.50] | 713.00 [613.25–732.50] | 848.00 [770.25–898.75] |
| MPV, fL | 8.95 [8.53–9.00] | 8.75 [8.32–8.95] | 8.60 [8.40–8.88] |
| T3 — 30 days of age | | | |
| Body weight, kg | 86.00 [85.25–88.25] | 90.50 [89.00–94.25] | 88.50 [83.50–93.50] |
| Serum iron, mg/dL | 82.50 [41.50–114.50] | 68.00 [53.75–85.25] | 37.50 [29.50–58.25] |
| HCT, % | 25.40 [22.95–28.53] | 23.80 [20.00–32.33] | 22.85 [17.77–27.62] |
| HGB, g/dL | 8.90 [8.32–10.38] | 9.30 [8.40–10.50] | 8.35 [7.60–8.80] |
| MCV, fL | 33.10 [28.35–34.10] | 30.60 [27.07–35.48] | 33.25 [24.85–34.15] |
| MCH, pg | 10.65 [10.43–11.47] | 11.10 [10.83–11.38] | 10.90 [10.18–11.47] |
| MCHC, g/dL | 33.05 [31.45–38.77] | 36.40 [31.60–40.68] | 34.00 [31.90–41.20] |
| RDW, % | 39.60 [37.23–41.67] | 40.60 [40.12–41.48] | 40.60 [39.12–42.25] |

| | | | |
|---|---|---|---|
| Reticulocytes, K/μL | 1.00 [0.77–1.00] | 0.90 [0.23–1.20] | 0.80 [0.65–0.95] |
| Neutrophils, % | 47.10 [44.75–47.72] | 47.15 [45.70–53.10] | 50.40 [49.47–52.15] |
| Lymphocytes, % | 45.75 [44.40–46.35] | 42.85 [40.10–46.50] | 44.05 [38.38–44.85] |
| Monocytes, % | 6.65 [5.70–8.65] | 5.05 [3.80–5.62] | 4.85 [3.65–6.12] |
| Eosinophils, % | 0.15 [0.10–0.20] | 0.20 [0.12–0.20] | 0.10 [0.10–0.18] |
| Neutrophils, $10^9$/L | 5.87 [5.12–6.37] | 6.29 [6.07–6.60] | 5.69 [5.21–6.04] |
| Lymphocytes, $10^9$/L | 5.85 [5.38–6.12] | 5.51 [4.72–5.86] | 4.97 [4.56–5.17] |
| Monocytes, $10^9$/L | 0.86 [0.78–0.99] | 0.57 [0.48–0.68] | 0.48 [0.37–0.66] |
| Eosinophils, $10^9$/L | 0.01 [0.01–0.02] | 0.02 [0.01–0.03] | 0.01 [0.01–0.03] |
| Platelets, K/μL | 702.00 [565.00–760.25] | 629.00 [596.25–727.75] | 798.50 [592.25–1151.00] |
| MPV, fL | 8.65 [8.53–9.07] | 8.85 [8.43–9.05] | 8.70 [8.50–9.00] (n=5) |

Values are median [25th–75th percentile]. Each treatment group comprised six calves. Cell-specific sample sizes (n) are shown when data were missing.
Abbreviations: HCT, haematocrit; HGB, haemoglobin; MCV, mean corpuscular volume; MCH, mean corpuscular haemoglobin; MCHC, mean corpuscular haemoglobin concentration; RDW, red cell distribution width; MPV, mean platelet volume.

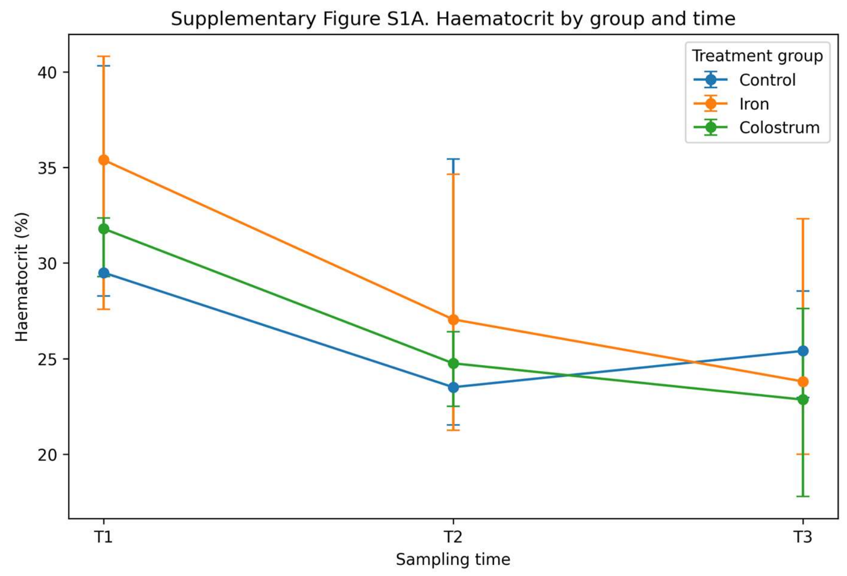


**Supplementary Figure S1A.**
Haematocrit (HCT) in Chianina calves assigned to the Control, Iron, and Colostrum groups at 48 h (T1), 14 days (T2), and 30 days (T3) of age. Data are presented as median and interquartile range (n = 6 calves per group).

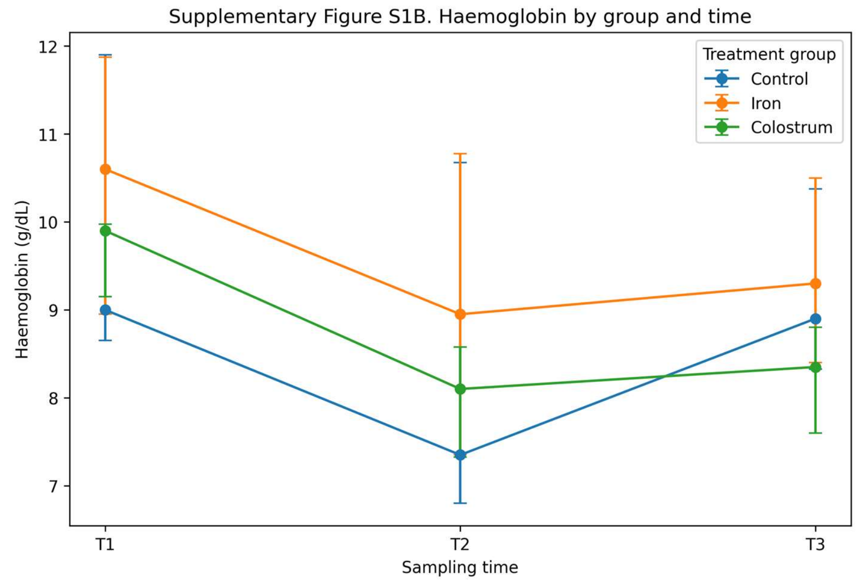


**Supplementary Figure S1B.**
Haemoglobin concentration (HGB) in Chianina calves assigned to the Control, Iron, and Colostrum groups at 48 h (T1), 14 days (T2), and 30 days (T3) of age. Data are presented as median and interquartile range (n = 6 calves per group).
